# A potential cure for tumor‐associated immunosuppression by *Toxoplasma gondii*

**DOI:** 10.1002/cnr2.1963

**Published:** 2023-12-18

**Authors:** Narges Lotfalizadeh, Soheil Sadr, Solmaz Morovati, Mohammadhassan Lotfalizadeh, Ashkan Hajjafari, Hassan Borji

**Affiliations:** ^1^ Department of Pathobiology, Faculty of Veterinary Medicine Ferdowsi University of Mashhad Mashhad Iran; ^2^ Division of Biotechnology, Department of Pathobiology, School of Veterinary Medicine Shiraz University Shiraz Iran; ^3^ Board Certificate Oral and Maxillofacial Radiologist North Khorasan University of Medical Sciences (NKUMS) Bojnurd Iran; ^4^ Department of Pathobiology, Faculty of Veterinary Medicine Islamic Azad University, Science and Research Branch Tehran Iran

**Keywords:** cancer, cancer immunotherapy, pathogen, *Toxoplasma gondii*

## Abstract

**Background:**

Recently, immunotherapy has become very hopeful for cancer therapy. Cancer treatment through immunotherapy has excellent specificity and less toxicity than conventional chemoradiotherapy. Pathogens have been used in cancer immunotherapy for a long time. The current study aims to evaluate the possibility of *Toxoplasma gondii* (*T. gondii*) as a probable treatment for cancers such as melanoma, breast, ovarian, lung, and pancreatic cancer.

**Recent findings:**

Nonreplicating type I uracil auxotrophic mutants of *T. gondii* can stimulate immune responses against tumors by reverse immunosuppression at the cellular level. *T. gondii* can be utilized to research T helper 1 (Th1) cell immunity in intracellular infections. Avirulent *T. gondii* uracil auxotroph vaccine can change the tumor's immunosuppression and improve the production of type 1 helper cell cytokines, i.e., Interferon‐gamma (IFN‐γ) and Interleukin‐12 (IL‐12) and activate tumor‐related Cluster of Differentiation 8 (CD8+) T cells to identify and destroy cancer cells. The *T. gondii* profilin protein, along with *T. gondii* secreted proteins, have been found to exhibit promising properties in the treatment of various cancers. These proteins are being studied for their potential to inhibit tumor growth and enhance the effectiveness of cancer therapies. Their unique mechanisms of action make them valuable candidates for targeted interventions in ovarian cancer, breast cancer, pancreatic cancer, melanoma, and lung cancer treatments.

**Conclusion:**

In summary, the study underscores the significant potential of harnessing *T. gondii*, including its diverse array of proteins and antigens, particularly in its avirulent form, as a groundbreaking approach in cancer immunotherapy.

## 1 INTRODUCTION

During cancer development, normal cells transform into cells that do not stop reproducing.^1^ The immune system usually eliminates transformed cells and identifies them to prevent cancer metastasis. However, in some conditions, the removal of tumors becomes ineffective.^2^ Although essential in numerous situations, conventional treatment methods such as surgery and chemotherapy frequently have significant drawbacks and constraints.^3^ Healthy cells, as well as cancerous cells, can be damaged by chemotherapy.^4^ When it comes to advanced‐stage cancers, surgery may not be a viable option, even if it is effective for localized tumors.^5^ The advent of immunotherapy, on the other hand, has instilled fresh optimism in cancer treatment.^6^ Through immunotherapy, the immune system is stimulated to fight off cancer cells.^7^ In comparison with conventional treatments, this approach is more specific in targeting tumor cells and offers less toxicity.^8^ By utilizing the potential of the immune system, immunotherapy seeks to enhance the body's inherent ability to combat cancer cells.^9^, ^10^ Using these therapies, the immune system is stimulated or manipulated so that cancer cells can be recognized and destroyed more effectively by the immune system. In addition to having shown remarkable success in treating some types of cancer, immunotherapy has proven to be variable in its efficiency among different patients, and research continues to be conducted to improve its results.^11^, ^12^

Employing pathogenic microorganisms in cancer immunotherapy is not new. *Serratia* spp,^13^ *Streptococcus* spp,^14^ *Listeria monocytogenes*,^15^ *Escherichia coli*,^16^ *Lactobacillus casei*,^17^ *Oncolytic virus*,^18^ *Talimogene laherparepve*,^19^ Newcastle disease virus,^20^ yellow fever vaccine 17D,^21^ *Trypanosoma cruzi*,^22^ *Echinococcus granulosus*,^23^, ^24^ and *Trichinella spiralis*,^25^ have been shown to eradicate tumor cells.^26^ *Toxoplasma gondii* (*T. gondii*) can regulate innate immune cells by invading myeloid cells, typically producing powerful Th1 immune responses.^27^ *T. gondii* secretes a collection of effector molecules during the invasion, allowing it to control the host cells.^28^, ^29^ The parasite even attacks adjacent cells that are reached but not invaded. For example, *T. gondii* activates the ROP16‐mediated STAT3 and STAT6 signaling pathways in macrophages, leading to downregulation of IL‐12 production and induction of arginase 1, respectively.^30^, ^31^ *T. gondii* release polymorphic rhoptry protein kinase ROP18 as well.^32^ This protein protects the parasite‐containing vacuoles against the host's innate immunity mediated by IFN‐activated GTPases.^29^, ^33^, ^34^ *T. gondii*, as a eukaryotic microbe, does not produce significant toxins or other toxic molecules, unlike many prokaryotic microbes.^35^ A recent study developed a stable and safe single‐copy variant of *T. gondii as* an uracil auxotroph (*cps*) strain.^36^ During the invasion of mammalian cells without uracil, the virulence of the *cps* strain was significantly reduced in both normal and severely immunodeficient mice.^37^ This engineered uracil auxotroph elicited strongly polarized Th1 host responses that could enhance the activity of innate immune cells in the tumor microenvironment and lead to tumor regression.

In an analysis of 150 cancer patients and 120 normal people without cancer, it was observed that individuals with low levels of anti‐*T. gondii* antibodies may have a more favorable prognosis.^38^ This suggests that an asymptomatic *T. gondii* infection has the potential to stimulate the immune system's response against tumors. Transcriptomics data indicated that *T. gondii* influences gene regulation, particularly in pathways associated with breast cancer, colorectal cancer, and non‐small cell lung cancer.^39^ This interference may ultimately impede tumor growth. Further, *T. gondii* infection appears to inhibit tumor growth and its invasion in many cancers. Hypoxia, avascular necrosis, suppression of tumor angiogenesis, and induction of Th1 immunity have all been proposed as potential mechanisms why *T. gondii* has antitumor effects.^40^, ^41^ The underlying reason for these effects appears to stem from the similarity between the molecular signaling pathways regulated by the parasite and those disrupted during the development of cancer. As an example, *T. gondii* infection can transform natural killer (NK) cells into ILC1 cells within the tumor microenvironment (TME).^42^ This phenomenon signifies the presence of a cell population with immune memory capabilities that can circulate throughout the body and continue producing IFN‐g.

**FIGURE 1:**
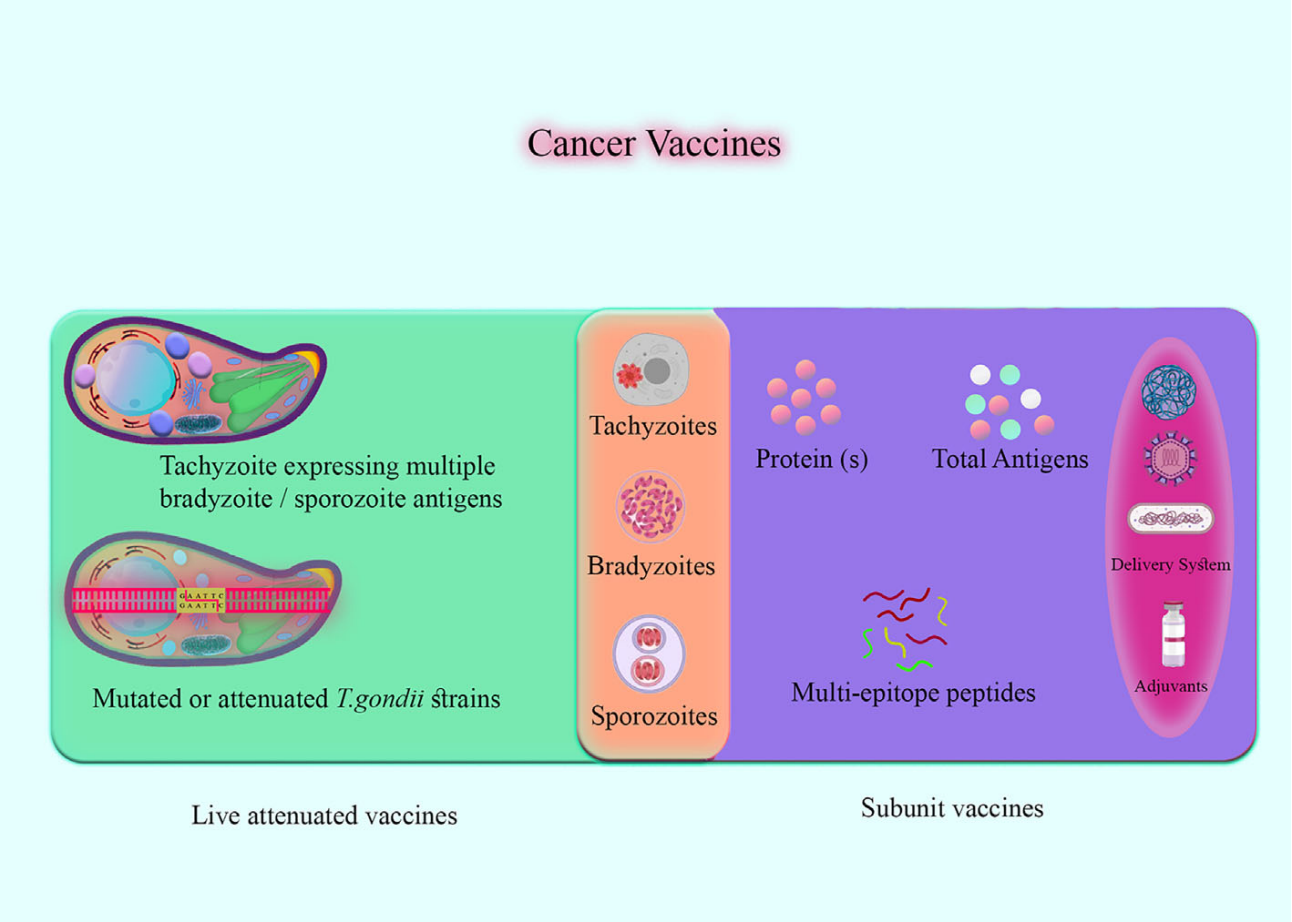
An overview of various approaches to developing toxoplasmosis vaccines is presented in this figure. The infection process of *Toxoplasma gondii* consists of three distinct stages: tachyzoites, bradyzoites (cysts), and sporozoites (oocysts). Regardless of the stage of *T. gondii*, multiple bradyzoite/sporozoite antigens can be expressed on tachyzoites to enhance live vaccines' immunogenicity. Recombinant immunogenic proteins and *T. gondii* extracts are used to generate vaccines containing *T. gondii* antigens from an array of species. Delivery systems and adjuvants are crucial for improving subunit vaccine effectiveness by enhancing antigen availability and enhancing immune responses. It is necessary to conduct further research to identify specific *T. gondii* epitopes that target CD4+, CD8+, and B cells. An epitope vaccine that elicits robust T and B cell responses needs to be designed rationally based on this knowledge. A vaccine based on these epitopes would broaden the spectrum of immune responses induced by antigens to a wide range.

Recombinant immunogenic proteins and *T. gondii* extracts are used to generate vaccines containing *T. gondii* antigens from an array of species (Figure 1). To investigate the potential immunotherapeutic benefits of *T. gondii* in cancer treatment, the current study has compiled a summary detailing the mechanisms through which *T. gondii* infection demonstrates an anti‐tumor impact. Furthermore, the present study focused on attenuated strains of *T. gondii* and its proteins and the activation of anti‐tumor signaling within the host triggered by *T. gondii* infection. The current review aimed to evaluate *T*. gondii as a possible treatment for melanoma, breast, ovarian, lung, prostate, and pancreatic cancers. Furthermore, the potential of combining cancer immunotherapy and nanotechnology is explained.

## 2 ROLE OF CYTOKINES AND IMMUNE RESPONSES IN ANTICANCER MECHANISMS

When infected with pathogens, dendritic cells (DCs) improve their ability to present antigens, secrete anti‐tumor cytokines, and activate innate immune responses in TMEs.^43^ Various immune cells, including antigen‐presenting cells (APCs), NK cells, CD4 and CD8+ lymphocytes, and B‐cells, have been found to produce and release IFN‐γ.^44^, ^45^ This cytokine has the ability to trigger macrophages and the expression of major histocompatibility complex (MHC) class I or II, as well as APC's costimulatory molecules.^46^

**FIGURE 2:**
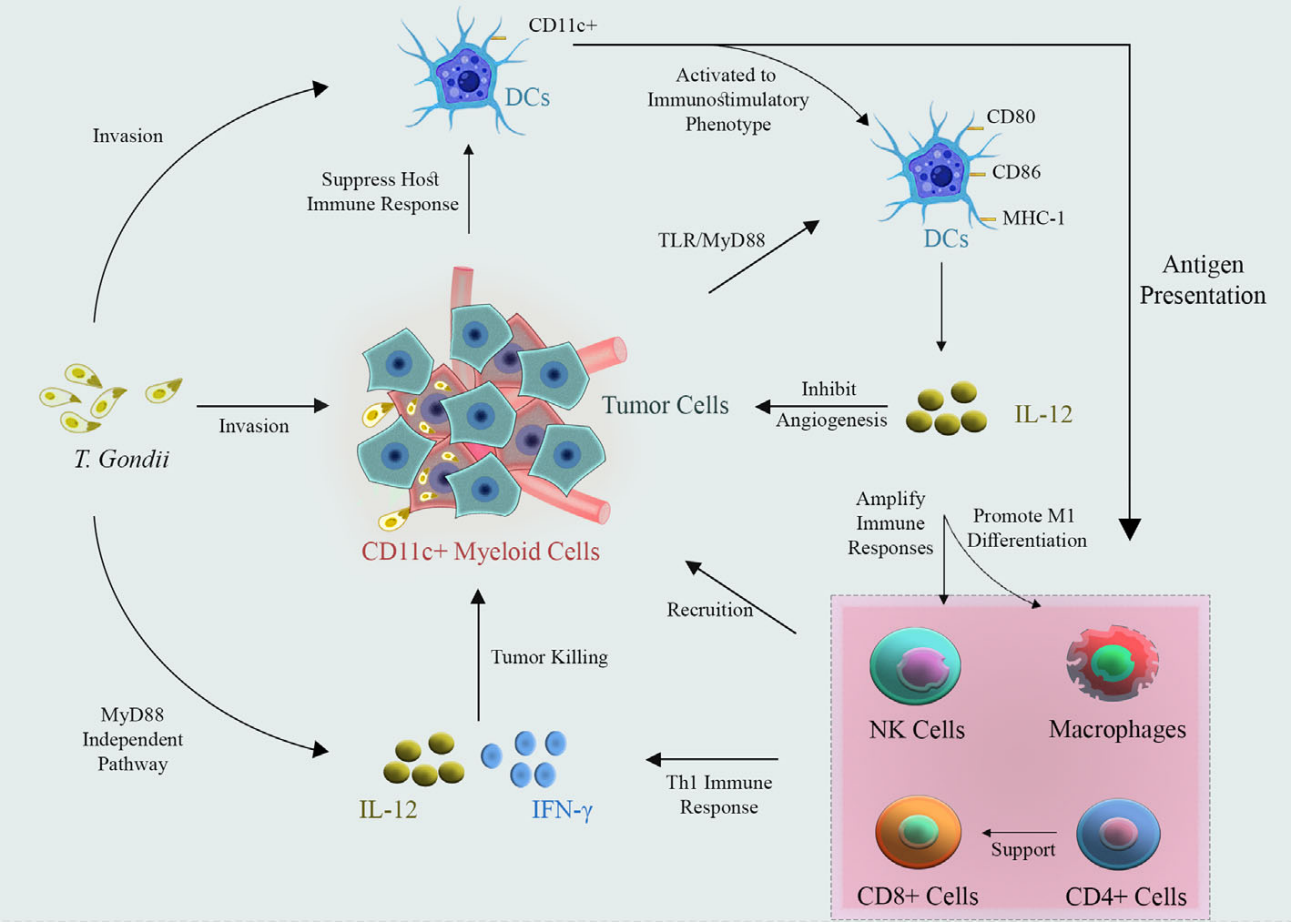
This figure provides an overview of different categories of *Toxoplasma gondii* vaccines used in research and development. The represented vaccine types include inactivated vaccines, excretory‐secretory antigen vaccines, live attenuated vaccines, subunit vaccines, DNA vaccines, epitope vaccines, and mRNA vaccines. Each category represents a distinct approach to *T. gondii* vaccine development, with unique characteristics and potential applications.

IFN‐γ and IFNα have several similar biological functions, including inducing signaling pathways that inhibit cell proliferation and promote the expression of MHC class I molecules.^47^, ^48^ Additionally, IFNα impedes the advancement of cancer. It induces apoptosis in a caspase‐dependent manner, resulting in anti‐angiogenic effects and enhancing the cytotoxicity and survival of NK cells, ultimately leading to tumor cell apoptosis.^49^ Another significant cytokine, IL‐12, is predominantly secreted by APCs in response to pathogens. It plays a crucial role in promoting cell‐mediated immunity and has been shown to drive the polarization of Th1 lymphocytes, increase NK and T lymphocyte activity, and elevate the expression of IFN‐γ.^50^ In in vivo, infection of *T. gondii* with macrophages can suppress the growth of tumor cells in mice with immune‐deficient systems, and *in‐vitro* infection can activate macrophages to kill cancer cells.^51^, ^52^, ^53^ The effectiveness of Non‐Replicating *Toxoplasma* Uracil Auxotrophs (NRTUAs) in combating tumors is diminished when CD8+ and NK cells are depleted, while the presence of CD4+ cells does not have the same impact.^54^ Studies have indicated that CD8+ T cells and CD8+ T cells are essential for achieving best anti‐cancer responses. Following NRTUA vaccination, there is a rapid increase in CD19+ B cells as well as CD3+ and CD8+ T cells at the site of inoculation.^55^ Subsequently, a vigorous Th1 immune response was initiated, demonstrating the substantial potential of these cells in triggering a mutually beneficial immune response against tumors. All of these findings provide evidence that the suppression of tumors by NRTUAs relies on intact immunity, particularly the CD8‐dependent immunity. In addition to triggering CD8+ T cell‐dependent immunity, NRTUA infection also stimulates Th2 humoral immunity against cancer cells.^56^ In a murine model of pancreatic cancer (Pan02), NRTUA treatment stimulates the production of tumor‐specific IgG. Additionally, CD4+ T cells have been found to have an important function in the response to a subsequent challenge with Pan02.^57^ Overall, an enduring CD8 + ‐dependent immune response may be established with NRTUA therapy. An infection with *T. gondii* activates innate immunity to produce IL‐12, which promotes the T cells and NK cells' anti‐tumor responses by facilitating the polarization of M1 macrophages, inducing the expression of IFN‐g, and inhibiting angiogenesis.^58^, ^59^ Mice that lack functional DCs exhibit a notable shortage of IL‐12 and are more susceptible to *T. gondii*.^60^ Moreover, the IL‐12 triggered by *T. gondii* relies on the activation of CD8+ T cells through Toll‐like receptor (TLR) signaling.^61^ A significant elevation in the serum levels of IL‐12p40 and IL‐12p70 has been observed in mice with ovarian cancer, melanoma, and pancreatic cancer after administration of NRTUAs as vaccination.^54^ The treatment with RH‐Dcps, which activates myeloid cells, lost its ability to fight tumors in mice that lacked IL‐12p40 or IL‐12p35.^26^ This suggests that the production of IL‐12 by NRTUA‐stimulated cells is crucial for anti‐cancer immunity. Of note, there are two distinct immunological phases observed in the treatment of melanoma. In the initial stage, the presence of host IL‐12 is not necessary, but in the subsequent phase, it needs to be expressed by DCs and macrophages.^62^ While secretion of IL‐12 which is influenced by the presence of MYD88, is indispensable in resisting *T. gondii* infection, it is not essential for eliciting an anti‐cancer response in instances of melanoma and ovarian cancer.^63^ An effective anti‐tumor response is dependent on IL‐12 in pancreatic cancer. A lack of IL‐12 in the body may make it difficult to eliminate malignant tumors and *T. gondii* infection (Figure 2).

## 3 *Toxoplasma gondii* PROFILIN PROTEIN

Actin‐binding *T. gondii* profilin (Tgprofilin) with the molecular weight of 14–19 kDa was found in Soluble *T. gondii* antigens (STAg).^64^ It enhances the immune response and lessens the pathogenic effects of bacteria, viruses, and other diseases‐causing agents.^65^ The profilin‐like protein (TgPLP) has a molecular weight of 17.5 kD and a high degree of similarity to Tgprofilin.^66^, ^67^ The distinction between Tgprofilin and TgPLP is not always clear in some researches. As a result, TgPLP is currently considered a component of Tgprofilin. Tgprofilin serves multiple functions, including contributing to *T. gondii*'s movement and attacking host cells. Moreover, through the activation of MyD88, it also stimulates the production of IL‐12 by acting as an agonist for TLR11.^66^ As a supplementary mechanism, TgPLP stimulates MyD88 signaling pathway during autologous whole‐tumor‐cell vaccine therapy. Macrophages derived from bone marrow express more antigen‐presenting cell biomarkers as a result of this activation. As a result, the production of IL‐12 is enhanced, improving the macrophages' ability to engulf cancer cells.^68^ Despite profilin or STAg administration, IFN‐g deficient mice (IFN‐g −/−) were unable to effectively suppress pancreatic tumor growth. This suggests that pancreatic cancer treatment requires DCs and IFN‐g.

## 4 *Toxoplasma gondii* SECRETED PROTEINS

**FIGURE 3:**
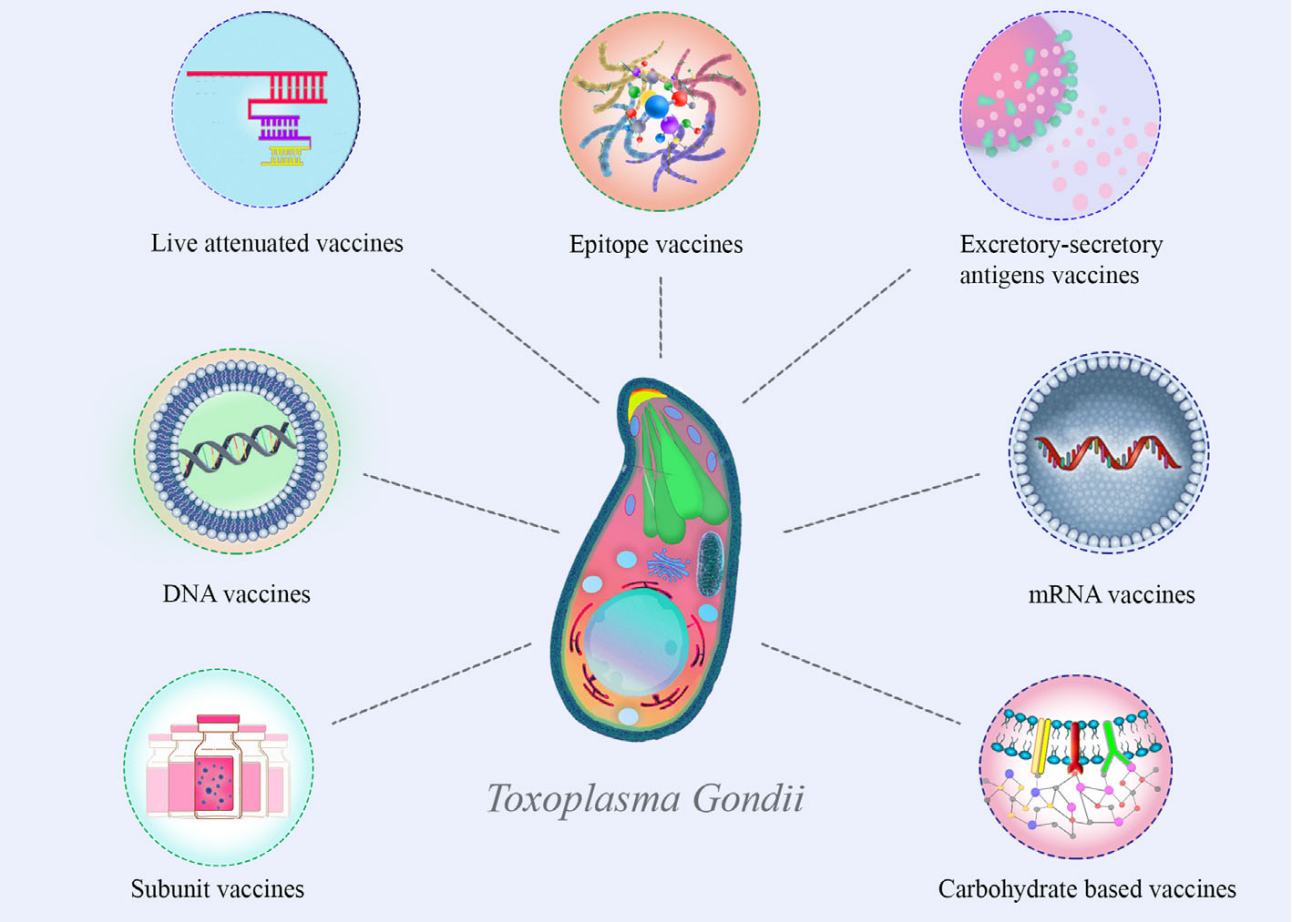
This figure illustrates the key processes involved in reversing immunosuppression for tumor therapy through *Toxoplasma gondii* vaccination. Myeloid cells in the tumor stroma are initially immunosuppressive, leading to reduced dendritic cell antigen‐presenting capacity and diminished immune responses. *T. gondii* infection, either systemically or within tumor tissues, alleviates myeloid cell immunosuppression. This triggers the activation of DCs, which modifies the tumor microenvironment by secreting IL‐12, promoting Th1 immune responses. *T. gondii* antigens, potent IL‐12 inducers via the MyD88 signaling pathway, play a crucial role. IL‐12 influences natural killer (NK) cells, CD4+, and CD8+ T lymphocytes, acting as a Th1 response stimulator and an angiogenesis inhibitor. Consequently, CD8+ T cells, NK cells, and macrophages are recruited to the tumor tissue, collaborating with their secreted IL‐12 and IFN‐γ to eliminate tumor cells effectively.

A tachyzoite culture medium contains excretory/secretory proteins (ESP) released by *T. gondii*.^69^ In individuals who have been previously infected, these proteins contain elements that attach specifically to serum antibodies, as well as components that act as proteases.^70^ As with TLA components, ESPs pose the primary candidates for potential vaccines with anti‐tumor properties.^71^ Various cell lines, including MCF‐7 breast cancer cells, K562 erythroleukemic cells, and DU145 prostate cancer cells, exhibit apoptosis induced by *T. gondii* ESP.^72^ These cells were exposed to varying concentrations of *T. gondii* tachyzoites during the mid‐exponential phase. The findings revealed a remarkable dose‐dependent inhibitory impact of the parasite on cancer cell development. Moreover, the expression of p53 protein in A549 tumor cells was increased while the expression of anti‐apoptotic Bcl‐2 gene was down‐regulated following treatment with *T. gondii*. Additionally, *T. gondii* ESP prevents Lewis lung carcinoma, B16F10 melanoma, and prostate cancer cells from growing. It has been shown that the administration of ESP to mice after subcutaneous injection of Lewis lung cancer cells and B16F10 melanoma cells in the right armpit resulted in a significant decrease in Treg of CD4 + CD25 + Foxp3+ and an increase in NK cells in the spleen.^73^ Furthermore, ESP‐treated subjects had significantly smaller tumors than control subjects.^74^ HPLC analysis confirms the presence of dense granule antigen 2 (GRA2) and GRA5, among other proteins, in ESP components.^75^ For effective invasion, *T. gondii* produces proteins sequentially from its secretory organelles, which include Microneme Proteins (MICs), Rhoptry Proteins (ROPs), and Gross Adhesion Proteins (GRAs).^76^, ^77^ Signaling pathways are also regulated by these proteins. To facilitate parasite proliferation and viability, *T. gondii* releases specific proteins that are attracted to the parasitophorous vacuole membrane (PVM).^78^ These proteins are then recognized by host cells, triggering an immune response against the tumor. The removal of PVM‐related proteins as well as intra‐vacuolar network‐related GRAs diminishes anti‐cancer immunity.^79^, ^80^ Specifically, when rop5 (Drop5) was abrogated, the anti‐cancer immunity in IFN‐g‐induced mouse fibroblasts was severely compromised.^81^ By introducing a new synthesized GRA8 peptide, the exporting of GRA8 to mitochondria, its interplay with deacetylase (sirtuin‐3), and controlling of mitochondrial function are enhanced.^82^ This enhancement ultimately results in higher effectiveness of mitochondria‐targeted therapies for HCT116 tumors. However, the removal of GRA3 and ROP12 does not hinder the anti‐cancer impact of the microorganism. Treatment of lung carcinoma, melanoma, and colon adenocarcinoma with a combination of *T. gondii* type I ΔGRA17 strain with the concentration of 10^6^ tachyzoites and 250 μg PD‐L1 inhibitors demonstrated notable efficacy.^83^ This approach enhances the innate and adaptive immune pathways. Zhu et al. discovered that intertumoral injection of ΔGRA17 enhanced CD8+ cytotoxic T‐cell infiltration, which ultimately resulted in tumor regression via upregulating IFN‐γ and TNF‐α. Indeed, it may suppress the expression of PD‐1 in CD8+ T cells and Foxp3 in CD4+ T cells, preventing immunosuppressive pathways in TME. They also demonstrated that intertumoral administration of ΔGRA17 led to the infiltration of immune cells in both injected and distant tumors. This significantly improved the survival of mouse models without any evidence of the recurrence of their disease. DNA damage and signals from oncogenes can stimulate the expression of gene p53. PVM transports dense granule‐secreted GRA16 to the nucleus, where it alters host gene transcription.^84^ GRA16 interacts with the host proteins such as protein phosphatase 2A and ubiquitin‐specific protease 7. This interaction allows GRA16 to affect the progression of the cell cycle and restore p53 function. Five specific sequences within the host cell's nucleus were involved in this restoration. Additionally, GRA16 enhances the effectiveness of irinotecan, a chemotherapy drug, by suppressing the activity of NF‐kB in lung H1299 cancer cells.^85^ It is known that NF‐kB plays a role in immunosurveillance of cancer cells. In addition, it controls transcription factor communication (e.g., TRAFs), regulating cell proliferation, apoptosis, and the release of inflammatory cytokines.^86^, ^87^ (Figure 3).

## 5 OVARIAN CANCER

Ovarian cancer is the deadliest female reproductive cancer.^88^ Due to its generally vague symptoms, this cancer is often not detected until it has reached an advanced stage, making effective treatment difficult.^89^ DCs are the most prevalent leukocyte subpopulation in ovarian carcinoma and develop a highly immunosuppressive tumor environment at tumor sites. The infiltration of immature CD11c^+^ dendritic cells in solid epithelial ovarian tumors positively contributed to releasing various proangiogenic and immunosuppressive mediators.^90^, ^91^ A previous study, found that ovarian cancer‐infiltrating DCs display a classical phenotype but express high levels of PD‐1 and B7‐H1 on their surface.^92^ These cells inhibit the accumulation and functioning of CD8^+^ T cells within the tumor microenvironment and block Nuclear Factor Kappa B (NF‐kB) activation. Therefore, any successful immunotherapy approach for ovarian cancer must overcome the tumor's immunosuppressive microenvironment.^93^ In this regard, several therapeutic interventions involving immune stimulation of CD11c^+^ cells have been examined.^94^, ^95^, ^96^ Enhancing the antigen presentation capacity of ovarian cancer‐infiltrating DCs and subsequent T‐cell‐driven antitumor immunity can be achieved by reversing CD11c^+^ MHC‐II^+^ from an immunosuppressive to an immunostimulatory phenotype.

Intertumoral treatment with nonreplicating *cps* strain of *T. gondii* is demonstrated to reverse the tumor‐associated immunosuppression of CD11c^+^ APCs into immunostimulatory status.^37^ While resistance to *T. gondii* depends on MyD88‐mediated IL‐12 production, it is not crucial for the anti‐tumor responses of ovarian cancer. Fox and colleagues showed that in addition to activating host cell invasion, uracil auxotrophic mutants of *T. gondii* provide efficient antitumor immunity against ID8DV ovarian cancer through the secretion of vacuole membrane (PVM) associated rhoptry effector proteins such as ROP5, ROP17, ROP18, ROP35 or ROP38, and intravacuolar network‐related dense granule proteins GRA2, GRA12, and GRA24.^78^ A previous study by Baird et al. 2013 conducted a study on established aggressive ID8‐Vegf ovarian tumors.^26^ They found that *cps* immunotherapy preferentially restored the antigen cross‐presentation ability of CD11c^+^ cells, primed new CD8^+^ T cell responses, and enhanced the function of suppressed CD8+ T cell responses. Tumor regression caused by *cps* therapy is associated with high‐level expression of T‐cell receptor costimulatory molecules CD80 and CD86 and IL‐12 and IFN‐γ, which are crucial for activating antitumor T‐cell immunity. Notably, vaccination with *cps* strain represents a lifelong CD8^+^ immunity, and its effectiveness is not affected by prior exposure to *T. gondii*, which is prevalent in humans. Treatment with *cps* is also accompanied by accumulating some splenocytes, including macrophages, B cells, NK cells, and CD4+ T cells, and decreasing the number of Foxp3+ CD4+ T regulatory cells in the tumor microenvironment.

## 6 BREAST CANCER

Women were diagnosed with breast cancer more frequently than men in 2020, with an estimated 2.3 million new patients.^97^ Cancerous cells that lack human epidermal growth factor receptor‐2 (HER2), estrogen receptor (ER), and progesterone receptor (PR) are referred to as triple‐negative breast carcinomas (TNBC).^98^ TNBC is the most prevalent malignant breast cancer subtype. It has the worst prognosis among all the carcinomas.^99^ These days, medical procedures, radiation treatment, and chemotherapy are vital choices for treating malignant breast cancer routinely, and chemotherapy continues to be the backbone of TNBC therapy.^100^ Even though many new treatments have been assessed, the impacts of treating TNBC remain poor, emphasizing the need for a novel technique for viable TNBC treatment.^101^ Cancer vaccines have attracted a legion of attention as a way forward for cancer treatment. Nevertheless, the unfortunate immunogenicity of cancer‐related antigens often fails to elicit a productive and safe response against the tumor.^102^ Several studies have reported unique anticancer immune responses in parasite‐infected patients due to cross‐reactivity.^103^, ^104^, ^105^ Thus, parasite antigens with high immunogenicity and significant epitope similarity with breast cancer antigens may stimulate a robust and safe response against many tumor cells.^105^, ^106^ *T. gondii* can trigger an immune response caused by the release of IFN‐γ and IL‐12, similar to those against breast cancer.^107^ Inoculating an attenuated uracil auxotroph *T. gondii* vaccine might inhibit breast cancer progression if the parasite infection affects tumor‐related factors.^51^ Scientists hypothesize that infection with *T. gondii* may influence the breast cancer signaling pathway.^108^ The anti‐tumor potential of the avirulent uracil auxotroph RH‐Δompdc mutant was experimentally evaluated by Xu et al.^108^ They reasoned that intratumoral administration of these engineered live attenuated parasites into 4T1 breast tumor caused tumor regression and decreased lung metastasis. Moreover, the tumor‐bearing mice experienced a more prolonged survival rate. It is believed that the IL‐12 signaling pathway regulates these results. Monocytes, the earliest immune cells that secrete IL‐12 during *T. gondii* infection, trigger the production of more IL‐12 from DCs and neutrophils. In turn, the IL‐12 enhances the influx of NK and CD8^+^ T cells and secretion of IFN‐γ in both serum and tumor microenvironment.^109^ The secreted IFN‐γ eventually promotes cell‐mediated immunity against tumors by inducing apoptosis in cancer cells and preventing angiogenesis in tumor tissue.^110^

## 7 PANCREATIC CANCER

Pancreatic ductal adenocarcinoma (PDAC) remains one of the deadliest cancers, with an average survival time of only 4 to 6 months after diagnosis.^111^ Despite advances in tumor microenvironment and improvements in the production of therapies and drugs, this cancer continues to pose a significant challenge.^112^ Thirty percent of patients have advanced local disease, and 50% show evidence of metastatic disease at the time of diagnosis.^113^ Despite some progress in improving survival rates, it is still necessary to develop additional therapies and drugs that can develop immunity to more effectively treat advanced pancreatic cancer in conjunction with existing therapies.^114^ New therapies that provide long‐term prevention against tumor recurrence and deadly metastatic pancreatic cancer will be essential to enhance patients' long‐term survival. A favorable prognosis is associated with circulating CD8^+^ T cells in pancreatic cancer.

Myeloid cells undergo fast reprogramming to suppress or be suppressed within the pancreatic tumor microenvironment in response to cancer.^115^ Attenuated *T. gondii* vaccine strain *cps* blocks immune suppression in pancreatic tumors by activating tumor‐dependent myeloid cells, which then trigger a tumor cell antigen‐specific CD8+ T‐cell immune response.^54^ In a similar fashion to ovarian cancer, the expression of costimulatory molecules CD80 and CD86 as well as IL12 is upregulated by tumor‐associated DCs and macrophages. Although systemic administration of IL12 causes substantial toxicity in cancer patients, IL‐12 secreted following cps therapy exhibits no side effects.^116^ Subsequently, CD8+ T cells perform their anti‐tumor benefits by recognizing the pancreatic tumor‐cell antigen and producing IFNγ within the TME. Unlike ovarian and melanoma cancers, cps treatment activates the MyD88 signaling pathway in pancreatic cancer cells.

According to a study by Sanders et al., 2016 et al., mice bearing Pan02 pancreatic cancer that received cps therapy significantly increased activated CD8+ T cell infiltration into the tumor microenvironment.^57^ In more details, the high amount of pancreatic tumor antigens released by CD8^+^ effector T cells during primary cps therapy can elicit the secretion of tumor‐specific IgG, along with long lasting CD4^+^ T cell memory. Of note, while the survival against the primary tumor does not depend on CD4^+^ T cells, the distribution and stability of circulating tumor‐specific IgG provide a protective effect against tumor re‐challenge. However, further research is needed to fully understand the relationship between memory immune cells and circulating antibodies in the mechanism of action of the cps mutant of *T. gondii* for cancer treatment.^54^, ^57^, ^117^

## 8 MELANOMA

Despite recent progress in treatment strategies, melanoma remains the most commonly lethal form of skin cancer.^118^ Cancer immunotherapies have therefore been studied as possible treatments for metastatic melanoma.^119^, ^120^ A study have shown that CD8^+^ T cells are effective in treating melanoma. Baird et al. investigated the anti‐ B16F10 melanoma effects of the attenuated strain of *T. gondii* (cps) by intertumoral injection.^62^ Their study represented the first successful use of immune‐based monotherapy against primary B16F10 tumors in mice, which led to tumor shrinkage. They found that *cps*‐based monotherapy elicited a potent in vivo anti‐tumor immunological response driven by IFNγ‐expressing CD8^+^ T cells. As the T cells were activated, the dermal melanoma tumor regressed and rarely reappeared. Besides local effectiveness, this treatment induced systemic and memory anticancer immune activity. The mice that survived showed vitiligo, a sign of good outcomes that suggest cps treatment generated melanocyte‐specific CD8^+^ T cell immune reactions.^62^ The systemic response made immunotherapy preferable to surgical excision that can eradicate occult metastatic lesions. To enhance efficacy, cps monotherapy can be combined with additional immunological strategies, such as adoptive T‐cell treatment. The maturation of T‐cells was mediated by IL‐12, and the CXCR3‐stimulating cytokines. Despite pancreatic tumor, the release of IFNγ by NK‐cells was crucial for the successful treatment of the B16 melanoma tumor model.^57^ Surprisingly, it is suggested that under the presence of cps, Th1 CD4^+^ T cells are no longer playing their normal role through classic Th1‐type cells. Instead, other cell types such as NK cells are stepping in. Indeed, like that of ID8 ovarian cancer, the therapeutic efficacy of *cps* therapy was not dependent on the MYD88 signaling pathway.^26^

In general, evidence demonstrates that *cps* therapy causes immediate and long‐lasting leukocyte infiltration of the tumor. There have been two distinct immunological phases throughout melanoma treatment. Myeloid, rather than lymphoid, cell recruitment occurs as the initial response to the treatment. In this case, phagocytes constitute 70%–80% of *T. gondii*‐invaded cells, while only 20%–30% of T cells and neutrophils are affected by the parasite during the beginning phase of the treatment.^121^ Finally, an increase in IFNγ expression by both CD8+ T cells and NK cells, as well as an increase in the number of CD8+ T cells, occurred during subsequent treatment stages, resulting in durable protection against tumor rechallenge. This represents a significant advancement in the field of melanoma immunotherapy.

## 9 LUNG CANCER

Lung cancer is responsible for one of the highest mortality rates from cancer‐related conditions worldwide.^122^, ^123^ This condition is described as the uncontrolled growth of lung cells, often associated with malignant tumor growth. Lung cancers are classified into non‐small cell lung cancers (NSCLC) and small cell lung cancers (SCLC).^124^ The most common form of lung cancer is non‐small cell lung cancer (NSCLC).^125^ The advancement of medical science has simplified lung cancer treatment, but delayed diagnosis, low response rates to treatment, and limited therapeutic options remain significant obstacles.^126^ Several conventional treatments are available for lung cancer, including radiation therapy, chemotherapy, surgery, targeted therapies, and immunotherapy.^127^ In lung cancer cases that are discovered and treated at an early stage, treatments can be quite effective; their effectiveness, however, may alter according to the type of the cancer and its stage.^128^ Managing this condition has become more challenging as drug resistance has evolved.

Using *T. gondii* as a treatment strategy for lung cancer could be a promising research direction. *T. gondii* was investigated in a murine model to determine whether it could counter lung cancer. Based on a study findings, *T. gondii* infection suppresses Lewis lung carcinoma (LLC) growth in mice, exhibiting considerable tumor‐suppressing activity.^129^

In another study, survival rates of mice given *T. gondii* Me49 strain orally (TG‐injected) exceeded those of mice that received LLC injections.^130^ Furthermore, mice injected with *T. gondii* and LLC cells (TG/LLC‐injected group) displayed even better survival rates. Some things were associated with this result, such as higher CD8+ T‐cell levels, enhancement of IFN‐γ mRNA expression rates, increased IgG2a levels in the serum, and increased cytotoxic T lymphocyte (CTL) activity. When these immune responses are triggered, there is a robust immune reaction against cancer. In addition, the study found that mice injected with TG/LLC and TG showed antiangiogenic properties, preventing angiogenesis (formation of new blood vessels), an essential process for tumor proliferation. Tumor growth inhibition occurs as a result of this compound's antiangiogenic properties. A further enhancement of antitumor effects in mice injected with TG/LLC was obtained when Quil‐A was added as an adjuvant. However, it's important to note that this treatment approach resulted in lower levels of CD4+ and CD8+ T‐cells, IgG1 and IgG2a serum titers, and IFN‐γ transcripts in mice that were injected with TG/LLC rather than TG alone. *T. gondii* infection appears to affect immunological responses and tumor microenvironment.

Another study examined the effects of an immunostimulant composed of formalin‐fixed *T. gondii* organisms (f‐Tp).^131^ F‐Tp was used in this experiment to inhibit the growth of tumors in C57BL/6 mice infected with *T. gondii* and presenting with LLC. The substances showed significant antitumor effects when f‐Tp was administered intradermally to mice with tumor cells. The tumor was reduced in size, and a longer life span was observed in the mice. f‐Tp induced a delayed‐type hypersensitivity (DTH) response directly correlated with its antitumor activity. Interestingly, f‐Tp was an antitumor even when injected directly into tumor sites at hours 1, 3, and 5 after tumor inoculation. Furthermore, live BCG injections in BCG‐sensitized mice only had a significant antitumor effect when injected with tumor cells. As a result of these findings, f‐Tp injection in mice with *T. gondii* infection could induce potent antitumor responses.

Using a mouse xenograft model, researchers discovered that GRA16‐transfected H1299 cells minimized tumor size.^85^ Protein phosphatase 2A‐B55 activates upon induction of the B55 regulatory subunit by GRA16, causing the GWL protein level to decrease, the ENSA phosphorylation to increase, and PP2A‐B55 function to be activated. Therefore, cell survival and cell cycle arrest were suppressed, as well as phosphorylation of AKT/ERK. GRA16 enhanced Irinotecan's anticancer properties, as it inhibited NF‐B activity and resulted in cell cycle arrest, thereby enhancing chemotherapeutic efficacy against NSCLC. The purpose of this study was to investigate the effects of *T. gondii* excretory‐secretory antigens (ESA) on CD4 + CD25+ Foxp3+ Treg cells in mice with LLC and to determine whether *T. gondii* ESA inhibits tumor growth. C57BL/6 mice were divided into PBS and Lewis groups, with LLCs injected into the latter group. A subgroup of these groups was then subdivided into those treated with ESA and those not treated with ESA. PBS and Lewis groups showed increased spleen coefficient and reductions in splenic Treg cells after ESA treatment. Additionally, ESA treatment for *T. gondii* retarded tumor growth, as evidenced by significantly smaller tumors in Lewis patients who received ESA treatment versus patients who did not. In mice with LLC, the ESA of *T. gondii* inhibited tumor growth and reduced the proportion of Treg cells.

## 10 FUTURE AND CHALLENGES

It is essential to understand that tumor‐associated immunosuppression hinders cancer treatment effectiveness.^132^ The full potential of *T. gondii* as a therapeutic agent for reinvigorating the immune response against tumors must be achieved by overcoming a number of challenges. For this novel approach to be translated into a safe and practical cure for cancer‐associated immunosuppression, researchers and clinicians must address several challenges and directions in the future.

### 10.1 Safety and toxicity

A therapeutic agent such as *T. gondii* should not lead to human health problems. Studies have shown that *T. gondii* can boost immune responses against tumors, though it is necessary to watch out for adverse effects. Uncontrolled parasite reproduction, inflammatory processes, and unintentional tissue damage might all contribute to the problem.^133^ Preclinical and clinical trials must be conducted with robust safety assessments to identify and mitigate these risks. In the future, the therapeutic window needs to meet its full potential and ensure the safety and efficacy of therapeutic interventions.

### 10.2 Immunological balance

The delicate balance between reactivating the immune system and avoiding autoimmune reactions must be achieved to reactivate the immune response.^134^ Therapy based on *T. gondii* must target tumor‐specific antigens while sparing healthy tissues. Innovations in immunomodulatory techniques and precise targeting mechanisms are therefore necessary. A balance must be maintained between inducing dangerous autoimmune reactions and providing effective treatments to achieve successful outcomes. *T. gondii* interacts with the immune system in many ways that must be fully understood. This interaction needs further research to understand how the parasite triggers immune responses and modulates tumor‐specific immunity. Comprehending these mechanisms can enhance therapeutic strategies and interventions at a molecular level.

### 10.3 Resistance and adaptation

The various treatment options for cancer are noted to lose effectiveness over time,^135^ and *T. gondii*‐based therapy is no exception. One of the crucial aspects of comprehending this therapy is anticipating how tumor cells can adapt and resist it. Combination therapies, treatment cycling, and other innovative methods are available for overcoming this resistance. Cancer research is constantly evolving, and keeping up with tumor resistance mechanisms can be difficult.^136^

### 10.4 Personalized medicine

Individuals react differently to *T. gondii*‐based therapy influenced by genetic, immunological, and environmental factors.^137^ To enhance therapeutic results, it is imperative to design individualized treatment plans tailored to the unique characteristics of each patient. Treatment regimens consist of determining biomarkers and other predictive indicators for guiding treatment choices.

### 10.5 Long‐term monitoring and follow‐up

To better understand how *T. gondii*‐based treatment impacts patients' general health and immune systems over time, prolonged monitoring and follow‐up studies are needed. Research on the effectiveness and durability of immune responses caused by the therapy and clinical trials for late‐onset side effects must be conducted. This type of research is crucial for evaluating therapy's safety and efficacy over the long term while offering comprehensive support once treatment is completed.

### 10.6 Combination therapies

A synergistic combination with other immunotherapy approaches or conventional cancer treatments is being explored to optimize the efficacy of *T. gondii*‐based therapy. It is challenging to optimize treatment sequences and combinations. Identifying therapies that complement each other and offer the most excellent chance of improving treatment outcomes needs to be studied. Adding other therapies to chemotherapy may enhance its effectiveness in combatting different cancer types and resistance mechanisms.

## 11 NANOTECHNOLOGY FOR PERFECT ANTIGEN DELIVERY

In recent years, nanotechnology, or the science and engineering of materials at the nanoscale, has become a rapidly advancing field that has the potential to revolutionize all sectors of human society, including healthcare.^138^ In diagnostics, treatment, drug delivery, and disease management, nanomaterials with dimensions between 1 and 100 nanometers offer distinct advantages over traditional medicine.^139^ The role of nanotechnology in targeted therapies has been one of nanotechnology's most significant contributions to medicine.^140^, ^141^ Traditional medical treatments often have limited selectivity and affect healthy tissues, leading to side effects and reduced efficacy.^142^ There is a wide range of nanoparticles available today for delivering drugs, genes, and imaging agents to specific cells or tissues on the basis of their size and structural properties, such as liposomes, dendrimers, and nanomicelles.^143^, ^144^ The ability to cross the blood–brain barrier makes nanoparticles particularly valuable in treating conditions that were previously difficult to treat, such as brain tumors.^145^ In addition, controlled‐release nanosystems can be used to extend the exposure of drugs, resulting in increased compliance among patients and reduced frequency of administration.^146^

With nanotechnology's capacity for manipulating materials at the nanoscale, breakthrough strategies addressing complicated cancer treatment issues are now possible due to this technology.^147^, ^148^, ^149^ A growing number of researchers are focusing on using nanoscale technology and nanocoatings to improve the ability of *T. gondii* antigens and proteins.^150^, ^151^ Nanotechnology can enhance antigen presentation by improving *T. gondii* antigen or protein uptake by antigen‐presenting cells, such as DCs.^152^ A variety of applications and advantages can be found in nanoscale technologies.^153^, ^154^ Nanoparticles possess unique properties that enable them to serve as carriers for *T. gondii* antigens.^155^ In some cases, researchers can enhance the bioavailability and longevity of these antigens by encapsulating them or coupling them with nanoparticles.^156^ Through this strategy, the immune system will be targeted more precisely, tumor‐specific antigens will be presented more effectively, and tumors will be attacked more effectively with an immune response.^157^

In vaccine production, adjuvants have a significant role, and nanoformulated adjuvants offer unique advantages.^158^, ^159^ Using nano‐sized adjuvants may enhance immune responses via stimulation of antigen‐presenting cells and stimulation of cytokine production together with antigens from *T. gondii*. A synergistic effect enhances the effectiveness of cancer vaccines based on *T. gondii*. Proteins delivery to target cells is facilitated by nanocarriers, such as liposomes, micelles, and dendrimers.^160^ By delivering these proteins precisely, they enhance their interaction with the host's immune system and are better able to function as cancer immunotherapies.^161^ Nanomaterials such as lipid bilayers or polymer shells can be used as coatings on *T. gondii* vaccines. These coatings serve various functions, including prolonging circulation times, preventing antigen degradation, and facilitating controlled release.^162^ The possibility of reducing off‐target effects and overall safety profiles can be enhanced, making the approach more effective.

**FIGURE 4:**
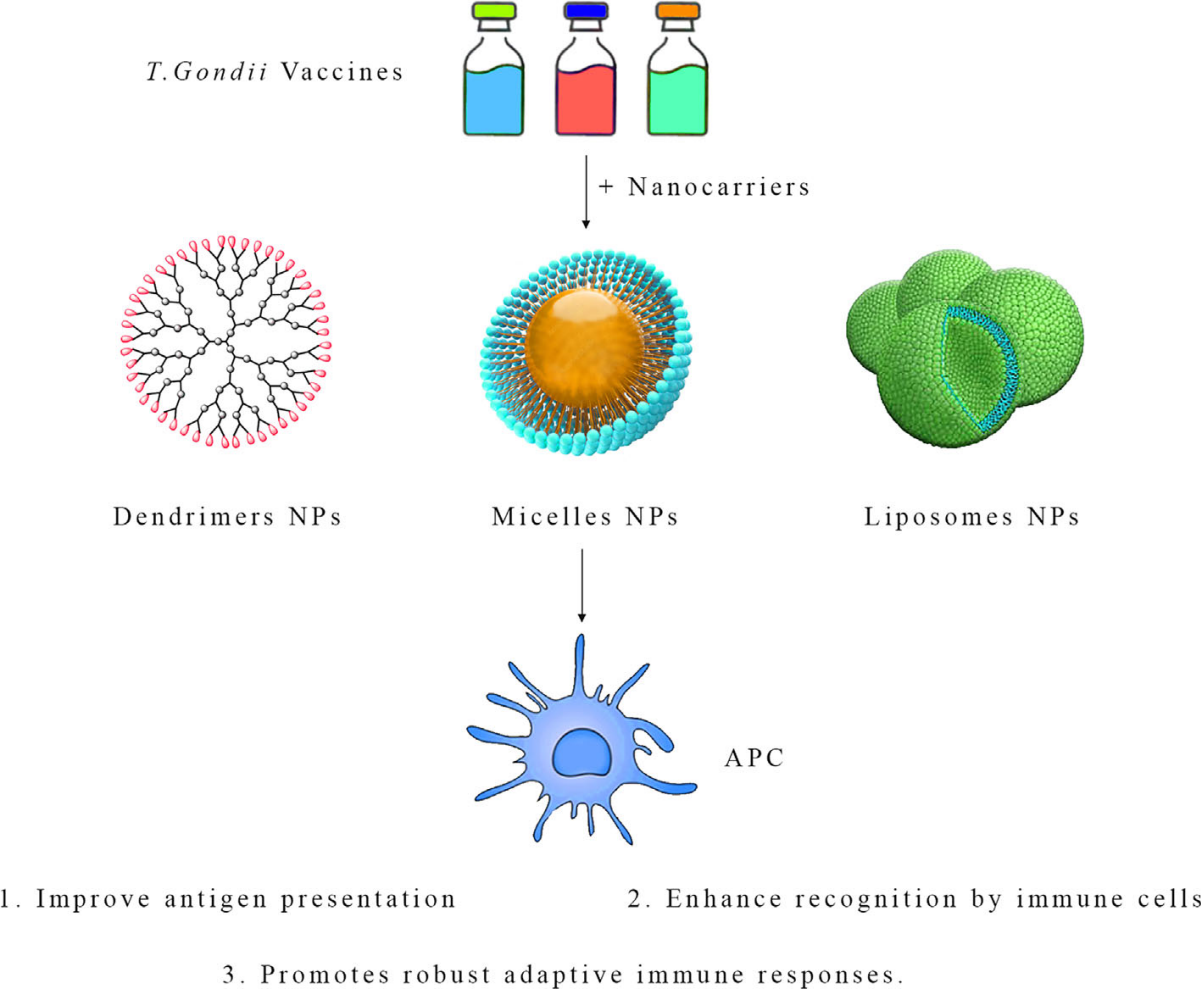
Dendrimers, micelles, and liposomes are excellent nano‐scale carriers for antigens of *Toxoplasma gondii*, as they can enhance the stimulation of the immune system.

The requirement for safety and biological compatibility is a crucial point to consider in nanotechnology applications. Their biocompatibility and toxicity must be thoroughly evaluated as a first step in establishing *T. gondii*‐based nano therapies as clinically viable. Nanotechnology combined with *T. gondii*‐based cancer immunotherapy is expected to be very beneficial in developing customized therapies. Clinical trials using customized nanocarriers and formulations allow clinicians to optimize treatment outcomes, ensuring that each patient receives an effective and highly personalized treatment plan (Figure 4).

## 12 CONCLUSION

Cancer immunotherapy has made remarkable advances in activating immune responses to eradicate tumors. An impressive list of cancers that *T. gondii* has been documented to alleviate includes melanoma, pancreatic, breast, lung, and ovarian cancers. Treatments that stimulate similar cytokines, chemokines, and immune responses to those used to treat cancer have a high effectiveness rate. Cancer immunotherapy has made remarkable advances in activating immune responses to eradicate tumors. An impressive list of cancers that *T. gondii* has been documented to alleviate include melanoma, pancreatic, breast, lung, and ovarian cancers. Treatments that stimulate similar cytokines, chemokines, and immune responses to those used to treat cancer have a high effectiveness rate. In the case of tumor‐associated immunosuppression, *T. gondii* may prove beneficial, but complex challenges must be overcome. Even though this innovative approach can reinvigorate the immune response against cancer, safety concerns, immunological balance, resistance mechanisms, and ethical concerns must be considered. Through personalized medicine, combination therapies, and global collaboration, there is still the possibility of improving treatment outcomes in the future. As well as optimizing *T. gondii*‐based therapies using nanotechnology, breakthroughs may be possible in this field. Cancer patients worldwide can find hope and healing by using this field's determination, collaboration, and innovation to find a cure for tumor‐associated immunosuppression despite hurdles.

## AUTHOR CONTRIBUTIONS

**Narges Lotfalizadeh:** Data curation (equal); formal analysis (equal); investigation (equal); writing – original draft (equal); writing – review and editing (equal). **Soheil Sadr:** Conceptualization (equal); data curation (equal); formal analysis (equal); investigation (equal); methodology (equal); validation (equal); visualization (equal); writing – original draft (equal); writing – review and editing (equal). **Solmaz Morovati:** Data curation (equal); investigation (equal); writing – original draft (equal); writing – review and editing (equal). **Mohammadhassan Lotfalizadeh:** Data curation (equal); methodology (equal); writing – original draft (equal); writing – review and editing (equal). **Ashkan Hajjafari:** Data curation (equal); software (equal); visualization (equal); writing – original draft (equal). **Hassan Borji:** Conceptualization (equal); methodology (equal); project administration (equal); resources (equal); supervision (equal); visualization (equal); writing – review and editing (equal).

## CONFLICT OF INTEREST STATEMENT

The authors declare no conflict of interest.

## ETHICS STATEMENT

Not applicable.

## Data Availability

The datasets generated during and/or analyzed during the current study are available from the corresponding author on reasonable request.

## References

[1] Sung H , Ferlay J , Siegel RL , et al. Global cancer statistics 2020: GLOBOCAN estimates of incidence and mortality worldwide for 36 cancers in 185 countries. CA Cancer J Clin. 2021;71(3):209‐249. doi:10.3322/caac.21660 33538338

[2] Cox TR . The matrix in cancer. Nat Rev Cancer. 2021;21(4):217‐238. doi:10.1038/s41568-020-00329-7 33589810

[3] Nandini D , Rao RS , Hosmani J , Khan S , Patil S , Awan KH . Novel therapies in the management of oral cancer: an update. Dis Mon. 2020;66(12):101036. doi:10.1016/j.disamonth.2020.101036 32594997

[4] Behranvand N , Nasri F , Zolfaghari Emameh R , et al. Chemotherapy: A double‐edged sword in cancer treatment. Cancer Immunol Immunother. 2022;71(3):507‐526. doi:10.1007/s00262-021-03013-3 34355266 PMC10992618

[5] Mastronicola R , Le Roux P , Casse A , et al. Current approaches to salvage surgery for head and neck cancer: a comprehensive review. Cancer. 2023;15(9):2625. doi:10.3390/cancers15092625 PMC10177213 37174091

[6] Peng Y , Chen F , Li S , et al. Tumor‐associated macrophages as treatment targets in glioma. Brain Sci Adv. 2020;6(4):306‐323. doi:10.26599/BSA.2020.9050015

[7] Chen Q , Chen M , Liu Z . Local biomaterials‐assisted cancer immunotherapy to trigger systemic antitumor responses. Chem Soc Rev. 2019;48(22):5506‐5526. doi:10.1039/C9CS00271E 31589233

[8] De Matos AL , Franco LS , McFadden G . Oncolytic viruses and the immune system: the dynamic duo. Mol Ther Methods Clin Dev. 2020;17:349‐358. doi:10.1016/j.omtm.2020.01.001 32071927 PMC7015832

[9] Iglesias‐Escudero M , Arias‐González N , Martínez‐Cáceres E . Regulatory cells and the effect of cancer immunotherapy. Mol Cancer. 2023;22(1):26. doi:10.1186/s12943-023-01714-0 36739406 PMC9898962

[10] Dwivedi R , Pandey R , Chandra S , Mehrotra D . Dendritic cell‐based immunotherapy: a potential player in oral cancer therapeutics. Immunotherapy. 2023;15(6):457‐469. doi:10.2217/imt-2022-0238 37013843

[11] Feeney OM , Gracia G , Brundel DH , et al. Lymph‐directed immunotherapy–Harnessing endogenous lymphatic distribution pathways for enhanced therapeutic outcomes in cancer. Adv Drug Deliv Rev. 2020;160:115‐135. doi:10.1016/j.addr.2020.10.002 33039497

[12] Kciuk M , Yahya EB , Mohamed Ibrahim Mohamed M , et al. Recent advances in molecular mechanisms of cancer immunotherapy. Cancer. 2023;15(10):2721. doi:10.3390/cancers15102721 PMC10216302 37345057

[13] Araghi A , Hashemi S , Sepahi AA , Faramarzi MA , Amin M . Purification and study of anti‐cancer effects of Serratia marcescens serralysin. Iran J Microbiol. 2019;11(4):320. doi:10.18502/ijm.v11i4.1470 31719964 PMC6829104

[14] Zhou S , Gravekamp C , Bermudes D , Liu K . Tumour‐targeting bacteria engineered to fight cancer. Nat Rev Cancer. 2018;18(12):727‐743. doi:10.1038/s41568-018-0070-z 30405213 PMC6902869

[15] Oladejo M , Paterson Y , Wood LM . Clinical experience and recent advances in the development of listeria‐based tumor immunotherapies. Front Immunol. 2021;12:642316. doi:10.3389/fimmu.2021.642316 33936058 PMC8081050

[16] Chowdhury S , Castro S , Coker C , Hinchliffe TE , Arpaia N , Danino T . Programmable bacteria induce durable tumor regression and systemic antitumor immunity. Nat Med. 2019;25(7):1057‐1063. doi:10.1038/s41591-019-0498-z 31270504 PMC6688650

[17] Ni D , Qing S , Ding H , et al. Biomimetically engineered demi‐bacteria potentiate vaccination against cancer. Adv Sci. 2017;4(10):1700083. doi:10.1002/advs.201700083 PMC5644226 29051851

[18] Hemminki O , Dos Santos JM , Hemminki A . Oncolytic viruses for cancer immunotherapy. J Hematol Oncol. 2020;13(1):1‐15. doi:10.1186/s13045-020-00922-1 32600470 PMC7325106

[19] Andtbacka RH , Kaufman HL , Collichio F , et al. Talimogene laherparepvec improves durable response rate in patients with advanced melanoma. J Clin Oncol. 2015;33(25):2780‐2788. doi:10.1200/JCO.2014.58.3377 26014293

[20] Ghorbankhani GA , Mohammadi A , Kazemipour N , et al. Apoptotic activity of Newcastle disease virus in comparison with nisin A in MDA‐MB‐231 cell line. Vet Res Forum. 2023;14(1):29‐37. doi:10.30466/vrf.2022.542258.3297 36816859 PMC9906615

[21] Aznar MA , Molina C , Teijeira A , et al. Repurposing the yellow fever vaccine for intratumoral immunotherapy. EMBO Mol Med. 2020;12(1):e10375. doi:10.15252/emmm.201910375 31746149 PMC6949490

[22] Sadr S , Ghiassi S , Lotfalizadeh N , Simab PA , Hajjafari A , Borji H . Antitumor mechanisms of molecules secreted by Trypanosoma cruzi in colon and breast cancer: A review. Anticancer Agents Med Chem. 2023;23(15):1710‐1721. doi:10.2174/1871520623666230529141544 37254546

[23] Sadr S , Borji H . Echinococcus granulosus as a promising therapeutic agent against triplenegative breast cancer. Curr Cancer Ther Rev. 2023;19(4):292‐297. doi:10.2174/1573394719666230427094247

[24] Asouli A , Sadr S , Mohebalian H , Borji H . Anti‐tumor effect of protoscolex hydatid cyst somatic antigen on inhibition cell growth of K562. Acta Parasitol. 2023;68:385‐392. doi:10.1007/s11686-023-00680-3 36991291

[25] Sadr S , Yousefsani Z , Ahmadi Simab P , Jafari Rahbar Alizadeh A , Lotfalizadeh N , Borji H . Trichinella spiralis as a potential antitumor agent: an update. World's Vet J. 2023;13(1):65‐74. doi:10.54203/scil.2023.wvj7

[26] Baird JR , Fox BA , Sanders KL , et al. Avirulent *Toxoplasma gondii* generates therapeutic antitumor immunity by reversing immunosuppression in the ovarian cancer microenvironment. Cancer Res. 2013;73(13):3842‐3851. doi:10.1158/0008-5472.CAN-12-1974 23704211 PMC3702636

[27] Khan IA , Moretto M . Immune responses to *Toxoplasma gondii* . Curr Opin Immunol. 2022;77:102226. doi:10.1016/j.coi.2022.102226 35785567

[28] Sanchez SG , Besteiro S . The pathogenicity and virulence of *Toxoplasma gondii* . Virulence. 2021;12(1):3095‐3114. doi:10.1080/21505594.2021.2012346 34895084 PMC8667916

[29] Ihara F , Nishikawa Y . *Toxoplasma gondii* manipulates host cell signaling pathways via its secreted effector molecules. Parasitol Int. 2021;83:102368. doi:10.1016/j.parint.2021.102368 33905814

[30] Cai Y , Chen H , Jin L , You Y , Shen J . STAT3‐dependent transactivation of miRNA genes following *Toxoplasma gondii* infection in macrophage. Parasit Vectors. 2013;6(1):1‐9. doi:10.1186/1756-3305-6-356 24341525 PMC3878672

[31] Denkers EY , Bzik DJ , Fox BA , Butcher BA . An inside job: hacking into Janus kinase/signal transducer and activator of transcription signaling cascades by the intracellular protozoan *Toxoplasma gondii* . Infect Immun. 2012;80(2):476‐482. doi:10.1128/IAI.05974-11 22104110 PMC3264314

[32] Hernández‐de‐Los‐Ríos A , Murillo‐Leon M , Mantilla‐Muriel LE , et al. Influence of two major *Toxoplasma gondii* virulence factors (ROP16 and ROP18) on the immune response of peripheral blood mononuclear cells to human toxoplasmosis infection. Front Cell Infect Microbiol. 2019;9:413. doi:10.3389/fcimb.2019.00413 31867288 PMC6904310

[33] Niedelman W , Gold DA , Rosowski EE , et al. The rhoptry proteins ROP18 and ROP5 mediate *Toxoplasma gondii* evasion of the murine, but not the human, interferon‐gamma response. PLoS Pathog. 2012;8(6):e1002784. doi:10.1371/journal.ppat.1002784 22761577 PMC3386190

[34] Chen Y , Yu M , Hemandez J , Li J , Yuan Z‐G , Yan H . Immuno‐efficacy of DNA vaccines encoding PLP1 and ROP18 against experimental *Toxoplasma gondii*infection in mice. Exp Parasitol. 2018;188:73‐78. doi:10.1016/j.exppara.2018.04.003 29626423

[35] Fox BA , Butler KL , Guevara RB , Bzik DJ . Cancer therapy in a microbial bottle: uncorking the novel biology of the protozoan *Toxoplasma gondii* . PLoS Pathog. 2017;13(9):e1006523. doi:10.1371/journal.ppat.1006523 28910406 PMC5599061

[36] Fox B , Sanders K , Bzik D . Non‐replicating *Toxoplasma gondii* reverses tumor‐associated immunosuppression. Oncoimmunology. 2013;2(11):e26296. doi:10.4161/onci.26296 24353916 PMC3862683

[37] Fox BA , Sanders KL , Chen S , Bzik DJ . Targeting tumors with nonreplicating *Toxoplasma gondii* uracil auxotroph vaccines. Trends Parasitol. 2013;29(9):431‐437. doi:10.1016/j.pt.2013.07.001 23928100 PMC3777737

[38] Seyedeh MS , Nahid S , Nahid M , Shima DP , Morteza Y , Hossein YD . Low titer of antibody against *Toxoplasma gondii* may be related to resistant to cancer. J Cancer Res Ther. 2015;11(2):305‐307. doi:10.4103/0973-1482.144638 26148590

[39] Lu G , Zhou J , Zhao YH , Li QL , Gao YY , Wang L . Transcriptome sequencing investigated the tumor‐related factors changes after *T. gondii* infection. Front Microbiol. 2019;10:181. doi:10.3389/fmicb.2019.00181 30792708 PMC6374557

[40] Caner A . *Toxoplasma gondii* could have a possible role in the cancer mechanism by modulating the host's cell response. Acta Trop. 2021;220:105966. doi:10.1016/j.actatropica.2021.105966 34023305

[41] Hunter CA , Yu D , Gee M , et al. Cutting edge: systemic inhibition of angiogenesis underlies resistance to tumors during acute toxoplasmosis. J Immunol. 2001;166(10):5878‐5881. doi:10.4049/jimmunol.166.10.5878 11342601

[42] Park E , Patel S , Wang Q , et al. *Toxoplasma gondii* infection drives conversion of NK cells into ILC1‐like cells. elife. 2019;8:e47605. doi:10.7554/eLife.47605 31393266 PMC6703900

[43] Zhao H , Wu L , Yan G , et al. Inflammation and tumor progression: signaling pathways and targeted intervention. Signal Transduct Target Ther. 2021;6(1):263. doi:10.1038/s41392-021-00658-5 34248142 PMC8273155

[44] Nelson MA , Ngamcherdtrakul W , Luoh S‐W , Yantasee W . Prognostic and therapeutic role of tumor‐infiltrating lymphocyte subtypes in breast cancer. Cancer Metastasis Rev. 2021;40(2):519‐536. doi:10.1007/s10555-021-09968-0 33963482 PMC8424653

[45] Ghorbaninezhad F , Alemohammad H , Najafzadeh B , et al. Dendritic cell‐derived exosomes: a new horizon in personalized cancer immunotherapy? Cancer Lett. 2023;562:216168. doi:10.1016/j.canlet.2023.216168 37031915

[46] Lanitis E , Dangaj D , Irving M , Coukos G . Mechanisms regulating T‐cell infiltration and activity in solid tumors. Ann Oncol. 2017;28:xii18‐xii32. doi:10.1093/annonc/mdx238 29045511

[47] Salmaninejad A , Valilou SF , Shabgah AG , et al. PD‐1/PD‐L1 pathway: basic biology and role in cancer immunotherapy. J Cell Physiol. 2019;234(10):16824‐16837. doi:10.1002/jcp.28358 30784085

[48] Wang H , Hu H , Zhang K . Overview of interferon: characteristics, signaling and anti‐cancer effect. Arch Biotechnol Biomed. 2017;1:1‐16. doi:10.29328/journal.hjb.1001001

[49] Vidal P . Interferon α in cancer immunoediting: from elimination to escape. Scand J Immunol. 2020;91(5):e12863. doi:10.1111/sji.12863 31909839

[50] Tokunaga R , Zhang W , Naseem M , et al. CXCL9, CXCL10, CXCL11/CXCR3 axis for immune activation–a target for novel cancer therapy. Cancer Treat Rev. 2018;63:40‐47. doi:10.1016/j.ctrv.2017.11.007 29207310 PMC5801162

[51] Chen J , Liao W , Peng H . *Toxoplasma gondii* infection possibly reverses host immunosuppression to restrain tumor growth. Front Cell Infect Microbiol. 2022;12:959300. doi:10.3389/fcimb.2022.959300 36118042 PMC9470863

[52] Zhou D , Yuan Z , Zhao F , et al. Modulation of mouse macrophage proteome induced by *Toxoplasma gondii* tachyzoites in vivo. Parasitol Res. 2011;109:1637‐1646. doi:10.1007/s00436-011-2435-z 21584632

[53] Sasai M , Pradipta A , Yamamoto M . Host immune responses to *Toxoplasma gondii* . Int Immunol. 2018;30(3):113‐119. doi:10.1093/intimm/dxy004 29408976

[54] Sanders KL , Fox BA , Bzik DJ . Attenuated *Toxoplasma gondii* stimulates immunity to pancreatic cancer by manipulation of myeloid cell populations. Cancer Immunol Res. 2015;3(8):891‐901. doi:10.1158/2326-6066.CIR-14-0235 25804437 PMC4526316

[55] Gigley JP , Fox BA , Bzik DJ . Cell‐mediated immunity to *Toxoplasma gondii* develops primarily by local Th1 host immune responses in the absence of parasite replication. J Immunol. 2009;182(2):1069‐1078. doi:10.4049/jimmunol.182.2.1069 19124750 PMC2615398

[56] Ding H , Wu S , Jin Z , et al. Anti‐tumor effect of parasitic protozoans. Bioengineering. 2022;9(8):395. doi:10.3390/bioengineering9080395 36004920 PMC9405343

[57] Sanders KL , Fox BA , Bzik DJ . Attenuated *Toxoplasma gondii* therapy of disseminated pancreatic cancer generates long‐lasting immunity to pancreatic cancer. Oncoimmunology. 2016;5(4):e1104447. doi:10.1080/2162402X.2015.1104447 27141388 PMC4839330

[58] Goldszmid RS , Dzutsev A , Trinchieri G . Host immune response to infection and cancer: unexpected commonalities. Cell Host Microbe. 2014;15(3):295‐305. doi:10.1016/j.chom.2014.02.003 24629336 PMC3996827

[59] Biswas SK , Mantovani A . Macrophage plasticity and interaction with lymphocyte subsets: cancer as a paradigm. Nat Immunol. 2010;11(10):889‐896. doi:10.1038/ni.1937 20856220

[60] Dupont CD , Christian DA , Hunter CA , eds. Immune response and immunopathology during toxoplasmosis. Seminars Immunopathol. Springer; 2012. doi:10.1007/s00281-012-0339-3 PMC3498595 22955326

[61] Pifer R , Yarovinsky F . Innate responses to *Toxoplasma gondii* in mice and humans. Trends Parasitol. 2011;27(9):388‐393. doi:10.1016/j.pt.2011.03.009 21550851 PMC3159709

[62] Baird JR , Byrne KT , Lizotte PH , et al. Immune‐mediated regression of established B16F10 melanoma by intratumoral injection of attenuated *Toxoplasma gondii* protects against rechallenge. J Immunol. 2013;190(1):469‐478. doi:10.4049/jimmunol.1201209 23225891 PMC3529845

[63] LaRosa DF , Stumhofer JS , Gelman AE , et al. T cell expression of MyD88 is required for resistance to *Toxoplasma gondii* . Proc Nattl Acad Sci USA. 2008;105(10):3855‐3860. doi:10.1073/pnas.0706663105 18308927 PMC2268781

[64] Rashidi S , Sánchez‐Montejo J , Mansouri R , et al. Mining the proteome of toxoplasma parasites seeking vaccine and diagnostic candidates. Animals. 2022;12(9):1098. doi:10.3390/ani12091098 35565525 PMC9099775

[65] Hedhli D , Dimier‐Poisson I , Judge JW , Rosenberg B , Mévélec MN . Protective immunity against Toxoplasma challenge in mice by coadministration of T. gondii antigens and Eimeria profilin‐like protein as an adjuvant. Vaccine. 2009;27(16):2274‐2281. doi:10.1016/j.vaccine.2009.01.100 19428842

[66] Yarovinsky F , Zhang D , Andersen JF , et al. TLR11 activation of dendritic cells by a protozoan profilin‐like protein. Science. 2005;308(5728):1626‐1629. doi:10.1126/science.1109893 15860593

[67] Yarovinsky F , Sher A . Toll‐like receptor recognition of *Toxoplasma gondii* . Int J Parasitol. 2006;36(3):255‐259. doi:10.1016/j.ijpara.2005.12.003 16476433

[68] Pyo K‐H , Lee Y‐W , Lim SM , Shin E‐H . Immune adjuvant effect of a *Toxoplasma gondii* profilin‐like protein in autologous whole‐tumor‐cell vaccination in mice. Oncotarget. 2016;7(45):74107‐74119. doi:10.18632/oncotarget.12316 27687589 PMC5342039

[69] Stryiński R , Łopieńska‐Biernat E , Carrera M . Proteomic insights into the biology of the most important foodborne parasites in Europe. Foods. 2020;9(10):1403. doi:10.3390/foods9101403 33022912 PMC7601233

[70] Keller P , Schaumburg F , Fischer SF , Häcker G , Groß U , Lüder CG . Direct inhibition of cytochrome c‐induced caspase activation in vitro by *Toxoplasma gondii* reveals novel mechanisms of interference with host cell apoptosis. FEMS Microbiol Lett. 2006;258(2):312‐319. doi:10.1111/j.1574-6968.2006.00241.x 16640590

[71] Rezaei F , Sharif M , Sarvi S , et al. A systematic review on the role of GRA proteins of *Toxoplasma gondii* in host immunization. J Microbiolog Met. 2019;165:105696. doi:10.1016/j.mimet.2019.105696 31442457

[72] Wu X , Sun L , Zhang L , Liu Z‐Q , Luo Q , Zhang L‐X . Impact of *Toxoplasma gondii* on the proliferation and apoptosis of tumor cell lines. Chin J Parasitol Parasit Dis. 2012;30(2):157‐159. 22908820

[73] Jiao YM , Zhang L , Ge YY , Liang YJ , Yong W . Effects of excreted/secreted antigens of *Toxoplasma gondii* on CD4+ CD25+ Foxp3+ T cells and NK cells of melanoma‐bearing mice. Chin J Schistosomiasis Control. 2011;23(3):301‐306. 22164498

[74] Yang MP , Goitsuka R , Ono K , Suzuki N , Hasegawa A . Effect of toxoplasma lysate antigen (TLA) on feline cytotoxicity against FeLV positive lymphoma cells. Nihon Juigaku Zasshi. 1990;52(4):735‐742. doi:10.1292/jvms1939.52.735 2167995

[75] Zenner L , Estaquier J , Darcy F , Maes P , Capron A , Cesbron‐delauw MF . Protective immunity in the rat model of congenital toxoplasmosis and the potential of excreted‐secreted antigens as vaccine components. Parasite Immunol. 1999;21(5):261‐272. doi:10.1046/j.1365-3024.1999.00229.x 10320624

[76] Wang Y , Yin H . Research advances in microneme protein 3 of *Toxoplasma gondii* . Parasit Vectors. 2015;8:1‐12. doi:10.1186/s13071-015-1001-4 26194005 PMC4509771

[77] Leroux L‐P , Dasanayake D , Rommereim LM , et al. Secreted *Toxoplasma gondii* molecules interfere with expression of MHC‐II in interferon gamma‐activated macrophages. Int J Parasitol. 2015;45(5):319‐332. doi:10.1016/j.ijpara.2015.01.003 25720921

[78] Fox BA , Sanders KL , Rommereim LM , Guevara RB , Bzik DJ . Secretion of rhoptry and dense granule effector proteins by nonreplicating *Toxoplasma gondii* uracil auxotrophs controls the development of antitumor immunity. PLoS Genet. 2016;12(7):e1006189. doi:10.1371/journal.pgen.1006189 27447180 PMC4957766

[79] Fox BA , Guevara RB , Rommereim LM , et al. *Toxoplasma gondii* parasitophorous vacuole membrane‐associated dense granule proteins orchestrate chronic infection and GRA12 underpins resistance to host gamma interferon. MBio. 2019;10(4):00589‐19. doi:10.1128/mBio.00589-19 PMC6606796 31266861

[80] Lockyer EJ , Torelli F , Butterworth S , et al. A heterotrimeric complex of Toxoplasma proteins promotes parasite survival in interferon gamma‐stimulated human cells. PLoS Biol. 2023;21(7):e3002202. doi:10.1371/journal.pbio.3002202 37459303 PMC10373997

[81] Zheng B , Lu S , Tong Q , Kong Q , Lou D . The virulence‐related rhoptry protein 5 (ROP5) of *Toxoplasma gondii* is a novel vaccine candidate against toxoplasmosis in mice. Vaccine. 2013;31(41):4578‐4584. doi:10.1016/j.vaccine.2013.07.058 23928460

[82] Kim J‐S , Lee D , Kim D , et al. *Toxoplasma gondii* GRA8‐derived peptide immunotherapy improves tumor targeting of colorectal cancer. Oncotarget. 2020;11(1):62‐73. doi:10.18632/oncotarget.27417 32002124 PMC6967779

[83] Zhu Y‐C , Elsheikha HM , Wang J‐H , et al. Synergy between *Toxoplasma gondii* type I ΔGRA17 immunotherapy and PD‐L1 checkpoint inhibition triggers the regression of targeted and distal tumors. J Immunother Cancer. 2021;9(11):e002970. doi:10.1136/jitc-2021-002970 34725213 PMC8562526

[84] Bougdour A , Durandau E , Brenier‐Pinchart M‐P , et al. Host cell subversion by toxoplasma GRA16, an exported dense granule protein that targets the host cell nucleus and alters gene expression. Cell Host Microbe. 2013;13(4):489‐500. doi:10.1016/j.chom.2013.03.002 23601110

[85] Seo S‐H , Kim S‐G , Shin J‐H , Ham D‐W , Shin E‐H . Toxoplasma GRA16 inhibits NF‐κB activation through PP2A‐B55 upregulation in non‐small‐cell lung carcinoma cells. Int J Mol Sci. 2020;21(18):6642. doi:10.3390/ijms21186642 32927892 PMC7554801

[86] Chakraborty C , Sharma AR , Sharma G , Lee S‐S . The interplay among miRNAs, major cytokines, and cancer‐related inflammation. Mol Ther Nucleic Acids. 2020;20:606‐620. doi:10.1016/j.omtn.2020.04.002 32348938 PMC7191126

[87] Habanjar O , Bingula R , Decombat C , Diab‐Assaf M , Caldefie‐Chezet F , Delort L . Crosstalk of inflammatory cytokines within the breast tumor microenvironment. Int J Mol Sci. 2023;24(4):4002. doi:10.3390/ijms24044002 36835413 PMC9964711

[88] Torre LA , Trabert B , DeSantis CE , et al. Ovarian cancer statistics, 2018. CA Cancer J Clin. 2018;68(4):284‐296. doi:10.3322/caac.21456 29809280 PMC6621554

[89] Lheureux S , Gourley C , Vergote I , Oza AM . Epithelial ovarian cancer. Lancet. 2019;393(10177):1240‐1253. doi:10.1016/S0140-6736(18)32552-2 30910306

[90] Magalhaes I , Fernebro J , Abd Own S , et al. Mesothelin Expression in Patients with High‐Grade Serous Ovarian Cancer Does Not Predict Clinical Outcome But Correlates with CD11c+ Expression in Tumor. Adv Ther. 2020;37(12):5023‐5031. doi:10.1007/s12325-020-01520-w 33052561 PMC7595982

[91] Hamanishi J , Mandai M , Iwasaki M , et al. Programmed cell death 1 ligand 1 and tumor‐infiltrating CD8+ T lymphocytes are prognostic factors of human ovarian cancer. Proc Natl Acad Sci USA. 2007;104(9):3360‐3365. doi:10.1073/pnas.0611533104 17360651 PMC1805580

[92] Krempski J , Karyampudi L , Behrens MD , et al. Tumor‐infiltrating programmed death receptor‐1+ dendritic cells mediate immune suppression in ovarian cancer. J Immunol. 2011;186(12):6905‐6913. doi:10.4049/jimmunol.1100274 21551365 PMC3110549

[93] Mellman I , Coukos G , Dranoff G . Cancer immunotherapy comes of age. Nature. 2011;480(7378):480‐489. doi:10.1038/nature10673 22193102 PMC3967235

[94] Conejo‐Garcia JR , Benencia F , Courreges M‐C , et al. Tumor‐infiltrating dendritic cell precursors recruited by a β‐defensin contribute to vasculogenesis under the influence of Vegf‐A. Nat Med. 2004;10(9):950‐958. doi:10.1038/nm1097 15334073

[95] Scarlett UK , Cubillos‐Ruiz JR , Nesbeth YC , et al. In situ stimulation of CD40 and toll‐like receptor 3 transforms ovarian cancer–infiltrating dendritic cells from immunosuppressive to immunostimulatory cells. Cancer Res. 2009;69(18):7329‐7337. doi:10.1158/0008-5472.CAN-09-0835 19738057 PMC2754806

[96] Cubillos‐Ruiz JR , Engle X , Scarlett UK , et al. Polyethylenimine‐based siRNA nanocomplexes reprogram tumor‐associated dendritic cells via TLR5 to elicit therapeutic antitumor immunity. J Clin Invest. 2009;119(8):2231‐2244. doi:10.1172/JCI37716 19620771 PMC2719935

[97] Ferlay J , Colombet M , Soerjomataram I , et al. Cancer statistics for the year 2020: an overview. Int J Cancer. 2021;149(4):778‐789. doi:10.1002/ijc.33588 33818764

[98] Waks AG , Winer EP . Breast cancer treatment: a review. JAMA. 2019;321(3):288‐300. doi:10.1001/jama.2018.19323 30667505

[99] Garrido‐Castro AC , Lin NU , Polyak K . Insights into molecular classifications of triple‐negative breast cancer: improving patient selection for treatment heterogeneity of triple‐negative breast cancer. Cancer Discov. 2019;9(2):176‐198. doi:10.1158/2159-8290.CD-18-1177 30679171 PMC6387871

[100] Britt KL , Cuzick J , Phillips K‐A . Key steps for effective breast cancer prevention. Nat Rev Cancer. 2020;20(8):417‐436. doi:10.1038/s41568-020-0266-x 32528185

[101] Harbeck N , Penault‐Llorca F , Cortes J , et al. Breast cancer. Nat Rev Dis Primers. 2019;5(1):1‐31. doi:10.1038/s41572-019-0111-2 31548545

[102] Arab A , Yazdian‐Robati R , Behravan J . HER2‐positive breast cancer immunotherapy: a focus on vaccine development. Arch Immunol Ther Exp. 2020;68(1):1‐18. doi:10.1007/s00005-019-00566-1 PMC7223380 31915932

[103] Yasen A , Wang M , Ran B , et al. Echinococcus granulosus protoscoleces promotes proliferation and invasion of hepatocellular carcinoma cells. Cytotechnology. 2021;73:13‐22. doi:10.1007/s10616-020-00437-0 33505110 PMC7817750

[104] Alvarez JP , Teneb J , Maldonado I , et al. Structural bases that underline Trypanosoma cruzi calreticulin proinfective, antiangiogenic and antitumor properties. Immunobiology. 2020;225(1):151863. doi:10.1016/j.imbio.2019.10.012 31732192

[105] Guan W , Zhang X , Wang X , Lu S , Yin J , Zhang J . Employing parasite against cancer: a lesson from the canine tapeworm Echinococcus granulocus. Front Pharmacol. 2019;10:1137. doi:10.3389/fphar.2019.01137 31607934 PMC6774290

[106] Callejas BE , Martinez‐Saucedo D , Terrazas LI . Parasites as negative regulators of cancer. Biosci Rep. 2018;38(5):BSR20180935. doi:10.1042/BSR20180935 PMC6200699 30266743

[107] Sasai M , Yamamoto M . Innate, adaptive, and cell‐autonomous immunity against *Toxoplasma gondii* infection. Exp Mol Med. 2019;51(12):1‐10. doi:10.1038/s12276-019-0353-9 PMC6906438 31827072

[108] Xu L‐Q , Yao L‐J , Jiang D , et al. A uracil auxotroph *Toxoplasma gondii* exerting immunomodulation to inhibit breast cancer growth and metastasis. Parasit Vectors. 2021;14(1):1‐14. doi:10.1186/s13071-021-05032-6 34895326 PMC8665513

[109] El Skhawy N , Eissa MM . Shedding light on a mysterious link between *Toxoplasma gondii* and cancer: a review. Exp Parasitol. 2023;250:108544. doi:10.1016/j.exppara.2023.108544 37149210

[110] Frucht DM , Fukao T , Bogdan C , Schindler H , O'Shea JJ , Koyasu S . IFN‐γ production by antigen‐presenting cells: mechanisms emerge. Trends Immunol. 2001;22(10):556‐560. doi:10.1016/S1471-4906(01)02005-1 11574279

[111] Han Y , Drobisch P , Krüger A , et al. Plasma extracellular vesicle messenger RNA profiling identifies prognostic EV signature for non‐invasive risk stratification for survival prediction of patients with pancreatic ductal adenocarcinoma. J Hematol Oncol. 2023;16(1):1‐19. doi:10.1186/s13045-023-01404-w 36737824 PMC9896775

[112] Kamisawa T , Wood LD , Itoi T , Takaori K . Pancreatic cancer. Lancet. 2016;388(10039):73‐85. doi:10.1016/S0140-6736(16)00141-0 26830752

[113] Ilic M , Ilic I . Epidemiology of pancreatic cancer. World J Gastroenterol. 2016;22(44):9694‐9705. doi:10.3748/wjg.v22.i44.9694 27956793 PMC5124974

[114] Rawla P , Sunkara T , Gaduputi V . Epidemiology of pancreatic cancer: global trends, etiology and risk factors. World J Oncol. 2019;10(1):10‐27. doi:10.14740/wjon1166 30834048 PMC6396775

[115] Arner EN , Rathmell JC . Metabolic programming and immune suppression in the tumor microenvironment. Cancer Cell. 2023;41:421‐433. doi:10.1016/j.ccell.2023.01.009 36801000 PMC10023409

[116] Atkins MB , Robertson MJ , Gordon M , et al. Phase I evaluation of intravenous recombinant human interleukin 12 in patients with advanced malignancies. Clin Cancer Res. 1997;3(3):409‐417. 9815699

[117] Bahwal SA , Chen JJ , Hao T , et al. Attenuated *Toxoplasma gondii* enhances the antitumor efficacy of anti‐PD1 antibody by altering the tumor microenvironment in a pancreatic cancer mouse model. J Cancer Res Clin Oncol. 2022;148:2743‐2757. doi:10.1007/s00432-022-04036-8 35556163 PMC11800998

[118] Shain AH , Bastian BC . From melanocytes to melanomas. Nat Rev Cancer. 2016;16(6):345‐358. doi:10.1038/nrc.2016.37 27125352

[119] Kuryk L , Bertinato L , Staniszewska M , et al. From conventional therapies to immunotherapy: melanoma treatment in review. Cancer. 2020;12(10):3057. doi:10.3390/cancers12103057 PMC7589099 33092131

[120] Domingues B , Lopes JM , Soares P , Pópulo H . Melanoma treatment in review. ImmunoTargets Ther. 2018;7:35‐49. doi:10.2147/ITT.S134842 29922629 PMC5995433

[121] Xue J , Jiang W , Chen Y , et al. Thioredoxin reductase from *Toxoplasma gondii*: an essential virulence effector with antioxidant function. FASEB J. 2017;31(10):4447‐4457. doi:10.1096/fj.201700008R 28687608

[122] Feng RM , Zong YN , Cao SM , Xu RH . Current cancer situation in China: good or bad news from the 2018 Global Cancer Statistics? Cancer Commun. 2019;39(1):1‐12. doi:10.1186/s40880-019-0368-6 PMC6487510 31030667

[123] Didkowska J , Wojciechowska U , Michalek IM , Caetano dos Santos FL . Cancer incidence and mortality in Poland in 2019. Sci Rep. 2022;12(1):10875. doi:10.1038/s41598-022-14779-6 35760845 PMC9237124

[124] Rajasegaran T , How CW , Saud A , Ali A , Lim JCW . Targeting inflammation in non‐small cell lung cancer through drug repurposing. Pharmaceutical. 2023;16(3):451. doi:10.3390/ph16030451 PMC10051080 36986550

[125] Padinharayil H , Varghese J , John MC , et al. Non‐small cell lung carcinoma (NSCLC): implications on molecular pathology and advances in early diagnostics and therapeutics. Genes Dis. 2023;10(3):960‐989. doi:10.1016/j.gendis.2022.07.023 37396553 PMC10308181

[126] Mamdani H , Matosevic S , Khalid AB , Durm G , Jalal SI . Immunotherapy in lung cancer: Current landscape and future directions. Front Immunol. 2022;13:823618. doi:10.3389/fimmu.2022.823618 35222404 PMC8864096

[127] Wang Y , Wang M , Wu HX , Xu RH . Advancing to the era of cancer immunotherapy. Cancer Commun. 2021;41(9):803‐829. doi:10.1002/cac2.12178 PMC8441060 34165252

[128] Howington JA , Blum MG , Chang AC , Balekian AA , Murthy SC . Treatment of stage I and II non‐small cell lung cancer: diagnosis and management of lung cancer: American College of Chest Physicians evidence‐based clinical practice guidelines. Chest. 2013;143(5):e278S‐e313S. doi:10.1378/chest.12-2359 23649443

[129] Yu‐Meng J , Zhi‐Yong T , Yu‐Jian C , et al. Inhibition of *Toxoplasma gondii* excretory‐secretory antigens on growth of murine Lewis lung carcinoma. Chin J Schistosomiasis Control. 2019;31(4):400. 10.16250/j.32.1374.2018269 31612675

[130] Kim J‐O , Jung S‐S , Kim S‐Y , et al. Inhibition of Lewis lung carcinoma growth by *Toxoplasma gondii* through induction of Th1 immune responses and inhibition of angiogenesis. J Korean Med Sci. 2007;22(Suppl):S38‐S46. doi:10.3346/jkms.2007.22.S.S38 17923753 PMC2694397

[131] Suzuki Y , Kobayashi A . Antitumor effect of intralesional injection with formalin‐fixed *Toxoplasma gondii* organisms on Lewis lung carcinoma in toxoplasma‐infected mice. Cancer Lett. 1985;25(3):247‐254. doi:10.1016/S0304-3835(15)30003-3 3971344

[132] Li M , He L , Zhu J , Zhang P , Liang S . Targeting tumor‐associated macrophages for cancer treatment. Cell Biosci. 2022;12(1):1‐13. doi:10.1186/s13578-022-00823-5 35672862 PMC9172100

[133] Gavrilescu LC , Denkers EY . Apoptosis and the balance of homeostatic and pathologic responses to protozoan infection. Infect Immun. 2003;71(11):6109‐6115. doi:10.1128/IAI.71.11.6109-6115.2003 14573625 PMC219574

[134] Aliberti J . Host persistence: exploitation of anti‐inflammatory pathways by *Toxoplasma gondii* . Nat Rev Immunol. 2005;5(2):162‐170. doi:10.1038/nri1547 15662369

[135] Gatenby RA , Brown JS . The evolution and ecology of resistance in cancer therapy. Cold Spring Harb Perspect Med. 2020;10(11):a040261. doi:10.1101/cshperspect.a040261 PMC7605238 33139405

[136] Arruebo M , Vilaboa N , Sáez‐Gutierrez B , et al. Assessment of the evolution of cancer treatment therapies. Cancer. 2011;3(3):3279‐3330. doi:10.3390/cancers3033279 PMC3759197 24212956

[137] Ahmadpour E , Babaie F , Kazemi T , et al. Overview of apoptosis, autophagy, and inflammatory processes in *Toxoplasma gondii* infected cells. Pathogens. 2023;12(2):253. doi:10.3390/pathogens12020253 36839525 PMC9966443

[138] Malik S , Muhammad K , Waheed Y . Nanotechnology: a revolution in modern industry. Molecules. 2023;28(2):661. doi:10.3390/molecules28020661 36677717 PMC9865684

[139] Chen G , Roy I , Yang C , Prasad PN . Nanochemistry and nanomedicine for nanoparticle‐based diagnostics and therapy. Chem Rev. 2016;116(5):2826‐2885. doi:10.1021/acs.chemrev.5b00148 26799741

[140] Sanna V , Pala N , Sechi M . Targeted therapy using nanotechnology: focus on cancer. Int J Nanomedicine. 2014;15:467‐483. doi:10.2147/IJN.S36654 PMC3896284 24531078

[141] Chaturvedi VK , Singh A , Singh VK , Singh MP . Cancer nanotechnology: a new revolution for cancer diagnosis and therapy. Curr Drug Metab. 2019;20(6):416‐429. doi:10.2174/1389200219666180918111528 30227814

[142] Parhi P , Mohanty C , Sahoo SK . Nanotechnology‐based combinational drug delivery: an emerging approach for cancer therapy. Drug Discov Today. 2012;17(17–18):1044‐1052. doi:10.1016/j.drudis.2012.05.010 22652342

[143] Zhu Y , Liao L . Applications of nanoparticles for anticancer drug delivery: a review. J Nanosci Nanotechnol. 2015;15(7):4753‐4773. doi:10.1166/jnn.2015.10298 26373036

[144] Wakaskar RR . General overview of lipid–polymer hybrid nanoparticles, dendrimers, micelles, liposomes, spongosomes and cubosomes. J Drug Target. 2018;26(4):311‐318. doi:10.1080/1061186X.2017.1367006 28797169

[145] Ferraris C , Cavalli R , Panciani PP , Battaglia L . Overcoming the blood–brain barrier: successes and challenges in developing nanoparticle‐mediated drug delivery systems for the treatment of brain tumours. Int J Nanomedicine. 2020;30:2999‐3022. doi:10.2147/IJN.S231479 PMC7201023 32431498

[146] Kashkooli FM , Soltani M , Souri M . Controlled anti‐cancer drug release through advanced nano‐drug delivery systems: static and dynamic targeting strategies. J Control Release. 2020;327:316‐349. doi:10.1016/j.jconrel.2020.08.012 32800878

[147] Gao S , Yang X , Xu J , Qiu N , Zhai G . Nanotechnology for boosting cancer immunotherapy and remodeling tumor microenvironment: the horizons in cancer treatment. ACS Nano. 2021;15(8):12567‐12603. doi:10.1021/acsnano.1c02103 34339170

[148] Li Y , Ayala‐Orozco C , Rauta PR , Krishnan S . The application of nanotechnology in enhancing immunotherapy for cancer treatment: current effects and perspective. Nanoscale. 2019;11(37):17157‐17178. doi:10.1039/C9NR05371A 31531445 PMC6778734

[149] Bockamp E , Rosigkeit S , Siegl D , Schuppan D . Nano‐enhanced cancer immunotherapy: immunology encounters nanotechnology. Cell. 2020;9(9):2102. doi:10.3390/cells9092102 PMC7565449 32942725

[150] Biswas P , Polash SA , Dey D , et al. Advanced implications of nanotechnology in disease control and environmental perspectives. Biomed Pharmacother. 2023;158:114172. doi:10.1016/j.biopha.2022.114172 36916399

[151] Assolini JP , Concato VM , Gonçalves MD , et al. Nanomedicine advances in toxoplasmosis: diagnostic, treatment, and vaccine applications. Parasitol Res. 2017;116:1603‐1615. doi:10.1007/s00436-017-5458-2 28477099

[152] Brito C , Lourenço C , Magalhães J , Reis S , Borges M . Nanoparticles as a delivery system of antigens for the development of an effective vaccine against *Toxoplasma gondii* . Vaccine. 2023;11(4):733. doi:10.3390/vaccines11040733 PMC10142924 37112645

[153] Azadi Y , Ahmadpour E , Ahmadi A . Targeting strategies in therapeutic applications of toxoplasmosis: recent advances in liposomal vaccine delivery systems. Curr Drug Targets. 2020;21(6):541‐558. doi:10.2174/1389450120666191023151423 31642775

[154] Król G , Fortunka K , Majchrzak M , et al. Metallic nanoparticles and core‐shell nanosystems in the treatment, diagnosis, and prevention of parasitic diseases. Pathogens. 2023;12(6):838. doi:10.3390/pathogens12060838 37375528 PMC10301874

[155] Yu Z , He K , Cao W , et al. Nano vaccines for T. gondii ribosomal P2 protein with nanomaterials as a promising DNA vaccine against toxoplasmosis. Front Immunol. 2022;13:839489. doi:10.3389/fimmu.2022.839489 35265084 PMC8899214

[156] Dimier‐Poisson I , Carpentier R , N'Guyen TTL , Dahmani F , Ducournau C , Betbeder D . Porous nanoparticles as delivery system of complex antigens for an effective vaccine against acute and chronic *Toxoplasma gondii* infection. Biomaterials. 2015;50:164‐175. doi:10.1016/j.biomaterials.2015.01.056 25736506

[157] Sadr S , Poorjafari Jafroodi P , Haratizadeh MJ , Ghasemi Z , Borji H , Hajjafari A . Current status of nano‐vaccinology in veterinary medicine science. Vet Med Sci. 2023;9:2294‐2308. doi:10.1002/vms3.1221 37487030 PMC10508510

[158] Zhou J , Kroll AV , Holay M , Fang RH , Zhang L . Biomimetic nanotechnology toward personalized vaccines. Adv Mater. 2020;32(13):1901255. doi:10.1002/adma.201901255 PMC6918015 31206841

[159] Das A , Ali N . Nanovaccine: an emerging strategy. Expert Rev Vaccines. 2021;20(10):1273‐1290. doi:10.1080/14760584.2021.1984890 34550859

[160] Tiwari H , Rai N , Singh S , et al. Recent advances in nanomaterials‐based targeted drug delivery for preclinical cancer diagnosis and therapeutics. Bioengineering. 2023;10(7):760. doi:10.3390/bioengineering10070760 37508788 PMC10376516

[161] Majidinia M , Mirza‐Aghazadeh‐Attari M , Rahimi M , et al. Overcoming multidrug resistance in cancer: recent progress in nanotechnology and new horizons. IUBMB Life. 2020;72(5):855‐871. doi:10.1002/iub.2215 31913572

[162] Yi Y , Yu M , Li W , Zhu D , Mei L , Ou M . Vaccine‐like nanomedicine for cancer immunotherapy. J Control Release. 2023;355:760‐778. doi:10.1016/j.jconrel.2023.02.015 36822241

